# Array Diagnosis and DOA Estimation for Coprime Array under Sensor Failures

**DOI:** 10.3390/s20092735

**Published:** 2020-05-11

**Authors:** Bing Sun, Chenxi Wu, Huailin Ruan

**Affiliations:** College of Electronic Engineering, National University of Defense Technology, Hefei 230037, China; wuchenxi19881201@126.com (C.W.); rhl_641111@163.com (H.R.)

**Keywords:** direction of arrival (DOA), coprime array, sensor failure, matrix reconstruction, redundant sensors

## Abstract

A coprime array of N sensors can achieve $O(N^{2})$ degrees of freedom (DOFs) by possessing a uniform linear array segment of size $O(N^{2})$ in the difference coarray. However, the structure of difference coarray is sensitive to sensor failures. Once the sensor fails, the impact of failure sensors on the coarray structure may decrease the DOFs and cause direction finding failure. Therefore, the direction of arrival (DOA) estimation of coprime arrays with sensor failures is a significant but challenging topic for investigation. Driven by the need for remedial measures, an efficient detection strategy is developed to diagnose the coprime array. Furthermore, based on the difference coarray, we divide the sensor failures into two scenarios. For redundant sensor failure scenarios, the structure of difference coarray remains unchanged, and the coarray MUSIC (CO-MUSIC) algorithm is applied for DOA estimation. For non-redundant sensor failure scenarios, the consecutive lags of the difference coarray will contain holes, which hinder the application of CO-MUSIC. We employ Singular Value Thresholding (SVT) algorithm to fill the holes with covariance matrix reconstruction. Specifically, the covariance matrix is reconstructed into a matrix with zero elements, and the SVT algorithm is employed to perform matrix completion, thereby filling the holes. Finally, we employ root-MUSIC for DOA estimation. Simulation results verify the effectiveness of the proposed methods.

## 1. Introduction

The direction of arrival (DOA) estimation problem plays an important role in array signal processing. In the modern electromagnetic environment, the sources are dense and complex. The conventional algorithms using uniform linear array (ULA) are difficult to perform accurate DOA estimation when there are more sources than sensors. To address this issue, the study in [1] proposed a coprime array structure to perform underdetermined DOA estimation. As a typical sparse array, the coprime array can resolve $O(N^{2})$ unrelated sources with N sensors compared to ULA. The coprime array has this advantage because they possess a uniform linear array segment of size $O(N^{2})$ in the difference coarray [2,3]. Recently, the research on the robustness of sparse arrays to sensor failures in [4,5] reveals that the coarray structure of coprime array is susceptible to sensors failure. The research of this paper was inspired by the literature [4,5]. Therefore, the research background in this paper is the same as that in [4,5], that is, the scenario where the sensor failed completely. Once the sensors fail, the impact of failure sensors on the difference coarray may decrease the degrees of freedom (DOFs) and cause performance degradation. In this scenario, it is important to quickly diagnose the coprime array and take remedial measures [6,7,8,9].

As shown in [4,5], the array robustness is determined by the number and location of failure sensors. The authors in [4,5] quantify the robustness of coprime array by introducing the *k*-essentialness of sensors and the *k*-essential family of arrays. This method is helpful for robust analysis, but it is relatively complicated to take remedial measures under sensor failures when *k* takes different values. The focus of this article is not to evaluate the robustness, but how to quickly classify the failure scenarios under the fact that sensor failure has already occurred, and the classification principle is to facilitate the subsequent remedial measures for DOA estimation. To perform DOA estimation for coprime array under sensor failures, we divide the sensor failures into two scenarios: redundant sensor failures and non-redundant sensor failures. If sensor failures cause the consecutive lags of difference coarray to change, the failure scenario is regarded as non-redundant sensor failures. Otherwise, the failure scenario is regarded as redundant sensor failures. This classification is convenient for the subsequent remedial measures for DOA estimation. In principle, this classification can be employed in array configurations with redundant sensors [10], such as uniform arrays, nested arrays, etc.

To address the issue of sensor failures, several methods have been proposed. In [11,12,13], the authors have studied the DOA estimation performance of the scenario where the sensor fails partially, but do not consider the scenario where the sensor fails completely. A neural network algorithm is presented in [14] to perform DOA estimation with sensor failures. However, a drawback of this method is that the initialization relies on trial and error method. The study in [15,16] proposes an iterative method for diagnosing failure sensors, but no remedial action was taken on the malfunctioning array. In [17,18,19], the authors restore the original pattern by optimizing the weight coefficients of the un-failed sensors. However, the maximum number of estimable signals cannot be recovered. The covariance matrix reconstruction method based on the difference coarray proposed in [20] uses lags to occupy failed sensors. However, this method is only applicable to the failure sensors in special positions and is not universal. In [21], the authors reconstruct a virtual array based on the Khatri-Rao product to handle failure array data, but this method only performs the DOA estimation of the redundant sensor failure scenario of the ULA.

To perform DOA estimation of coprime arrays under sensor failures, we advance a detection strategy to diagnose the coprime array. Based on the structure of difference coarray, we divide the sensor failures into two scenarios. For redundant sensor failure scenarios, the structure of difference coarray remains unchanged, and the coarray MUSIC (CO-MUSIC) algorithm is still applicable, which estimates source directions based on consecutive lags of difference coarray. For non-redundant sensor failures, the consecutive lags of difference coarray contain holes [22,23], which hinder the application of CO-MUSIC. Note that the sparsity-based approach [24,25] can work on the difference coarray of the malfunctioning array. However, the sparsity-based method requires discretization of the angular domain, which consequently leads to the disadvantage of base mismatch. To address this issue, Singular Value Thresholding (SVT) [26] algorithm is employed to fill the holes with covariance matrix reconstruction [27,28]. We reconstruct the covariance matrix into a matrix with zero elements, and employ the SVT algorithm to fill the zero elements. The equivalent result is that the holes of the non-uniform virtual linear array are filled to obtain an extended virtual uniform linear array, and the reconstructed high-dimensional covariance matrix is equivalent to the covariance matrix of an extended virtual uniform linear array. The number of lags in the virtual domain is larger than the number of actual physical sensors, so the method based on signal processing in the virtual domain can effectively improve the degree of freedom. Since the sensor spacing of the virtual uniform linear array meets the requirements of the Nyquist sampling theorem, the algorithm can realize the DOA estimation without phase ambiguity. SVT algorithm is easy to implement and effective in terms of computational cost and storage requirement. Finally, we employ root-MUSIC for DOA estimation. 

The main contribution of this paper is threefold:

- We advance a detection strategy to diagnose the failure sensors of the coprime array.
- By analyzing the structure of difference coarray under sensor failures, we divide the sensor failures into two scenarios. In principle, this classification can be employed in array configurations with redundant sensor, such as uniform arrays, nested arrays, etc.
- For non-redundant sensor failures, we reconstruct the covariance matrix into a matrix with zero elements, and employ the SVT algorithm to perform matrix completion to restore the DOA estimation performance.

The remainder of this paper is given as follows. In Section 2, we advance a detection strategy to diagnose the coprime array. Section 3 performs DOA estimation under sensor failures. Section 4 shows numerical simulations, and conclusions are drawn in Section 5.

## 2. Coprime Array Failure Sensor Detection and Classification

### 2.1. Coprime Array Model

**Figure 1:**
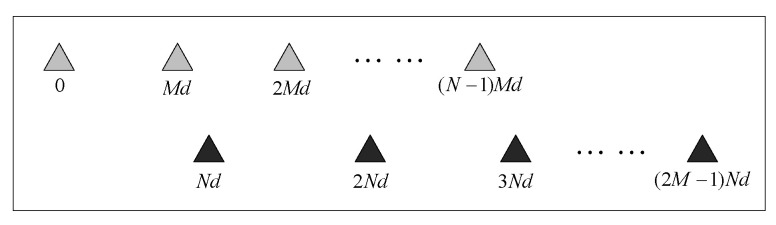
Illustration of the coprime array.

As shown in Figure 1, the coprime array consists of two uniform linear subarrays. Here M and N are coprime integers, d=λ/2 is half-wavelength of the signal, and λ is the wavelength. The first subarray consists of N sensors inter-element spacing Md, and the second subarray consists of 2M sensors inter-element spacing $Nd_{0}$. Two subarrays share the first sensor.

Assuming K uncorrelated far-field narrowband sources impinge on coprime array from the directions $\theta =[\theta_{1},\theta_{2},\cdots ,\theta_{K}]$, the coprime array received data are expressed as:

(1) $$x(t)=\sum_{k=1}^{K}a(\theta_{k})s_{k}(t)+n(t)=As(t)+n(t)$$

where $A=[a(\theta_{1}),a(\theta_{2}),\cdots ,a(\theta_{K})]$, and $a(\theta_{k})={\left[1,e^{j\frac{2\pi d}{\lambda }\sin\theta_{k}},\cdots ,e^{j\frac{2(2M+N-2)\pi d}{\lambda }\sin\theta_{k}}\right]}^{T}$ is the steering vector. $s(t)={\left[s_{1}(t),s_{2}(t),\cdots ,s_{K}(t)\right]}^{T}$ denotes K incident signals, $n(t)={\left[n_{1}(t),n_{2}(t),\cdots ,n_{2M+N-1}(t)\right]}^{T}$ is additive white Gaussian noise vector independent of signals.

The covariance matrix can be expressed as:

(2) $$\begin{array}{ll} R= & E\left\{x(t)x{\left(t\right)}^{H}\right\}\\ & =A\mathrm{diag}\left(\left[\sigma_{1}^{2},\sigma_{2}^{2},\ldots ,\sigma_{K}^{2}\right]\right)A^{H}+\sigma_{n}^{2}I \end{array}$$

where $\sigma_{n}^{2}$ is the noise variance, and the $\sigma_{k}^{2}$ denotes the *k*th input signal power.

In order to intuitively analyze the coprime array, the concept of the difference coarray is given, which is defined as:

(3) $$S=\{p_{i}-p_{j}\},\;p_{i},p_{j}\in \mathbb{P}$$

where ℙ denotes the position set of coprime array. $p_{i}$ is the position of the  i th sensor. S is the difference set of sensor positions, and the set including all the different values $p_{u}$ in S is defined as $S_{u}$. 

The coprime array in Figure 1 consists of 2M+N−1 sensors. From (3), the positions of each lag in the difference coarray are given by:

(4) $$\begin{array}{cc} S=\{\pm ( & Mnd-Nmd)\},\\ & 0\leq n\leq N-1,1\leq m\leq 2M-1 \end{array}$$

**Figure 2:**
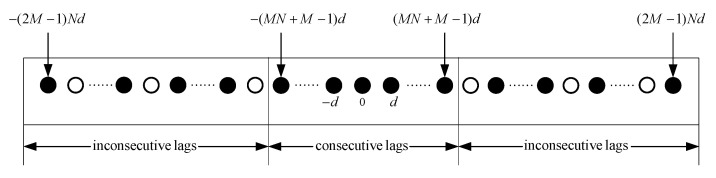
The difference coarray of coprime array.

From (4), the position distribution of lag in the difference coarray is shown in Figure 2.

As shown in Figure 2, the difference coarray can be divided into three parts, the first part is a virtual ULA with consecutive lags, and the other two parts are arrays of inconsecutive lags with holes. The virtual ULA with 2MN+2M−1 consecutive lags can employ subspace algorithms for DOA estimation, which is a conventional method for coprime array performing underdetermined DOA estimation and has good estimation performance. However, once the sensor fails, the difference coarray will change. The virtual ULA may have holes, which would invalidate conventional subspace methods. Different positions and numbers of failure sensors will also lead to different positions and numbers of holes.

### 2.2. Failure Sensor Detection and Classification

**Figure 3:**
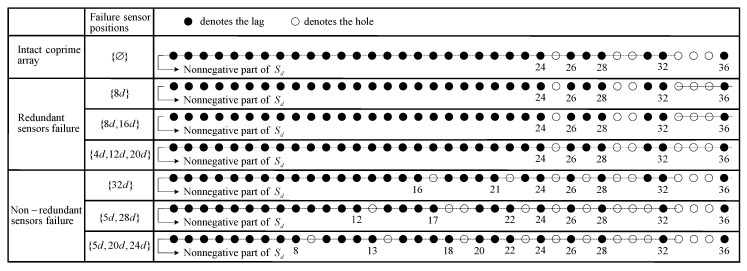
The nonnegative difference coarrays of the coprime array under different failure scenarios.

The pair of coprime integers are chosen as M=5 and N=4. The coprime array consists of 2M+N−1=13 sensors with the locations at [0,4d,5d,8d,10d,12d,15d,16d,20d,24d,28d,32d,36d]. The nonnegative difference coarrays of the co-prime array under different failure scenarios are shown in Figure 3. Three scenarios of redundant sensor failure and three scenarios of non-redundant sensor failure are listed. Comparing the redundant sensor failure scenarios, it can be seen that the number of failed redundant sensors has no effect on the difference coarray. Comparing the non-redundant sensor failure scenarios, it can be seen that the different numbers of failed non-redundant sensors have different effects on the difference coarray. Based on the above analysis, different failure scenarios have different impacts on the difference coarray. Therefore, it is important to diagnose the coprime array and classify the failure scenarios.

A malfunctioning sensor is considered to have failed completely. Failure of the i th array sensor is equivalent to all elements in row i of A set to zero. From (2), except R(i,i), the other elements in row i and column i of the R are zero. We use an array with N sensors for illustration, and suppose that the 2nd sensor is faulty. Then we can get:

(5) $$A=\left[\begin{array}{cccc} e^{j\frac{2\pi p_{1}}{\lambda }\sin\theta_{1}} & e^{j\frac{2\pi p_{1}}{\lambda }\sin\theta_{2}} & \cdots & e^{j\frac{2\pi p_{1}}{\lambda }\sin\theta_{k}}\\ 0 & 0 & \cdots & 0\\ e^{j\frac{2\pi p_{3}}{\lambda }\sin\theta_{1}} & e^{j\frac{2\pi p_{3}}{\lambda }\sin\theta_{2}} & \cdots & e^{j\frac{2\pi p_{3}}{\lambda }\sin\theta_{k}}\\ \vdots & \vdots & \ddots & \vdots \\ e^{j\frac{2\pi p_{N}}{\lambda }\sin\theta_{1}} & e^{j\frac{2\pi p_{N}}{\lambda }\sin\theta_{2}} & \cdots & e^{j\frac{2\pi p_{N}}{\lambda }\sin\theta_{k}} \end{array}\right]$$

Calculate the covariance matrix, we obtain:

(6) $$R=\left[\begin{array}{ccccc} \sum_{k=1}^{K}\sigma_{k}^{2}+\sigma_{n}^{2} & 0 & \sum_{k=1}^{K}\sigma_{k}^{2}e^{-jB} & \cdots & \sum_{k=1}^{K}\sigma_{k}^{2}e^{-jC}\\ 0 & \sigma_{n}^{2} & 0 & \cdots & 0\\ \sum_{k=1}^{K}\sigma_{k}^{2}e^{jB} & 0 & \sum_{k=1}^{K}\sigma_{k}^{2}+\sigma_{n}^{2} & \cdots & \sum_{k=1}^{K}\sigma_{k}^{2}e^{-jD}\\ \vdots & \vdots & \vdots & \ddots & \vdots \\ \sum_{k=1}^{K}\sigma_{k}^{2}e^{-jC} & 0 & \sum_{k=1}^{K}\sigma_{k}^{2}e^{jD} & \cdots & \sum_{k=1}^{K}\sigma_{k}^{2}+\sigma_{n}^{2} \end{array}\right]$$

where $B=\frac{2\pi (p_{3}-p_{1})}{\lambda }\sin\theta_{k}$, $C=\frac{2\pi (p_{N}-p_{1})}{\lambda }\sin\theta_{k}$, $D=\frac{2\pi (p_{N}-p_{3})}{\lambda }\sin\theta_{k}$. Under ideal assumptions, (6) shows that if there is only one non-zero element in a row and a column in theoretical covariance matrix R, then it can be determined that the sensor has failed. However, in practical applications, noise is not ideal additive white noise, which means that the covariance matrix of the noise is not diagonal matrix, and the noise is not independent of the signal. If sensor failure occurs in this practical application, there will not be a row and a column in the sample covariance matrix $\hat{R}$ with only one non-zero element. Instead, all elements in $\hat{R}$ are non-zero. Note that the elements in the $\hat{R}$ corresponding to the failed sensor are composed of the cross-correlation term between the signal and noise plus the noise cross-correlation term. Therefore, these elements are smaller than the others. In view of this, when the i th sensor fails, the sum of the absolute values of the elements in i th row is less than the average of other rows. The same goes for column i . We can diagnose coprime array as follows:

(7) $$\{\begin{array}{c} \;\hat{R}(i\;,\;:)\leq \eta \frac{1}{N+2M-1}\sum_{i=1}^{N+2M-1}\hat{R}(i\;,\;:)\\ \;\hat{R}(:\;,\;j)\leq \eta \frac{1}{N+2M-1}\sum_{j=1}^{N+2M-1}\hat{R}(:\;,\;j) \end{array}$$

where $\hat{R}(i\;,\;:)=\sum_{j=1}^{N+2M-1}\left|\hat{R}(i\;,\;j)\right|$ denotes the sum of absolute value of the elements in the i th row. $\hat{R}(:\;,\;j)=\sum_{i=1}^{N+2M-1}\left|\hat{R}(i\;,\;j)\right|$ denotes the sum of absolute value of the elements in the j th column. η is an empirical detection factor, and η∈( 0,1 ). If m exists so that $\hat{R}(m\;,\;:)$ and $\hat{R}(:\;,\;m)$ satisfy Equation (7), then we determine that the m th sensor of the coprime array fails.

The position of all failed sensors can be obtained through the above detection method. Then, for the non-failed sensors, we obtain the position difference set S through Equation (3), and further get the set $S_{u}$ including all the different values $p_{u}$ in S. Finally, we can obtain the number of consecutive lags from the set $S_{u}$ and record it as F. The difference coarray of intact coprime array possesses a virtual ULA with 2MN+2M−1 consecutive lags. If F=2MN+2M−1, we classify the failure scenario as redundant sensor failures, and if F<2MN+2M−1, non-redundant sensor failures.

The detection and classification in this paper is performed on the coprime array. In principle, the method can be employed in array configurations with redundant sensors, such as uniform arrays, nested arrays, etc.

## 3. DOA Estimation for Coprime Array Under Array Sensor Failures

### 3.1. Redundant Sensor Failures

For redundant sensor failures, the structure of difference coarray remains unchanged, and the CO-MUSIC algorithm is still applicable, which performs DOA estimation based on consecutive lags of difference coarray.

The coprime array in Figure 1 consists of 2M+N−1 sensors. From (2), we can get the covariance matrix as:

(8) $$\begin{array}{ll} R & =\left[\begin{array}{cccc} \sum_{k=1}^{K}\sigma_{k}^{2} & \sum_{k=1}^{K}\sigma_{k}^{2}e^{-jG} & \cdots & \sum_{k=1}^{K}\sigma_{k}^{2}e^{-jP}\\ \sum_{k=1}^{K}\sigma_{k}^{2}e^{jG} & \sum_{k=1}^{K}\sigma_{k}^{2} & \cdots & \sum_{k=1}^{K}\sigma_{k}^{2}e^{-jQ}\\ \vdots & \vdots & \ddots & \vdots \\ \sum_{k=1}^{K}\sigma_{k}^{2}e^{jP} & \sum_{k=1}^{K}\sigma_{k}^{2}e^{jQ} & \cdots & \sum_{k=1}^{K}\sigma_{k}^{2} \end{array}\right]+\sigma_{n}^{2}I\\ & =\left[\begin{array}{cccc} R(0) & R(-M) & \cdots & R(-(2M-1)N)\\ R(M) & R(0) & \cdots & R(M-(2M-1)N)\\ \vdots & \vdots & \ddots & \vdots \\ R((2M-1)N) & R((2M-1)N-M) & \cdots & R(0) \end{array}\right]+\sigma_{n}^{2}I \end{array}$$

where $G=\pi M\sin\theta_{k}$;$P=\pi (2M-1)N\sin\theta_{k}$;$Q=\pi ((2M-1)N-M)\sin\theta_{k}$; $\left\{R(p_{u})|p_{u}=-(2M-1)Nd,\cdots ,(2M-1)Nd\right\}$ is the elements of the covariance matrix.

The position set of each lag in the difference coarray becomes:

(9) $$S_{u}=\{-(2M-1)Nd,-(2M-2)Nd,\cdots ,(2M-1)Nd\}$$

Comparing Equations (8) and (9), it is clear that the elements of the covariance matrix corresponding to the positions of each lag. In practical applications, multiple sample covariance matrix elements corresponding to a lag are not equal, so we average the multiple elements corresponding to the same lag:

(10) $$\hat{R}(p_{u})=\frac{1}{\omega_{p}(p_{u})}\sum_{i=1}^{\omega_{p}(p_{u})}R_{i}\left(p_{u}\right)$$

where $\omega_{p}(p_{u})$ is the frequency of $p_{u}$ in the set S, and $R_{i}(p_{u})$ denotes ith element corresponding to a lag.

Assume that there are redundant sensor failures, and the number of non-failure sensors is $N_{1}$. The covariance matrix R of $N_{1}$ sensors can be given by Equation (2), and R is the $N_{1}\times N_{1}$ matrix. Then, we obtain the position difference set S through Equation (3), and further get the set $S_{u}$. The number of consecutive lags in the $S_{u}$ is 2MN+2M−1, and the nonnegative part is MN+M. According to the correspondence between the elements of the covariance matrix and the position of each lag, we reconstruct R into $R_{T}$ with the dimension (MN+M)×(MN+M):

(11) $$R_{T}(i,j)=\hat{R}\left((i-j)d\right)$$

The eigendecomposition of $R_{T}$ is:

(12) $$R_{T}=U_{S}\Sigma_{S}U_{S}^{H}+U_{n}\Sigma_{n}U_{n}^{H}$$

where $U_{S}$ denote the signal subspace eigenvectors, and $U_{n}$ is the noise subspace eigenvectors.

The MUSIC algorithm is used for spectral peak search:

(13) $$P(\theta_{i})=\frac{1}{a^{H}(\theta_{i})U_{n}U_{n}^{H}a(\theta_{i})}$$

The K largest peaks corresponding to the impinging sources.

### 3.2. Non-Redundant Sensor Failures

As Figure 3 shows, the non-redundant sensor failures results in a decrease of available consecutive lags, which may decrease DOFs and cause direction finding failure. To address this issue, the covariance matrix is reconstructed into a matrix with zero elements, and the SVT algorithm is employed to perform matrix completion, thereby filling the holes.

According to the correspondence between the elements of the covariance matrix and the position of each lag, we reconstruct and extend R into a high-dimensional $R_{T}$ with the dimension (2MN−N+1)×(2MN−N+1):

(14) $$R_{T}(i,j\;)=\{\begin{array}{cc} \hat{R}(p_{u}), & \;\mathrm{if}\;(i-j)\;d=p_{u},\\ \;0, & \mathrm{otherwise}, \end{array}$$

Some elements in the extended $R_{T}$ are zero, which is corresponding to the holes of the difference coarray. We perform matrix completion on the zero elements in $R_{T}$, thereby filling the holes. Take a coprime array with M=2, N=3 as an example. The coprime array consists of 2M+N−1=6 sensors at {0d,2d,3d,4d,6d,9d}. If non-redundant sensor at the {3d} position fails, the output covariance matrix is:

(15) $$R=\;\left[\begin{array}{ccccc} R(0) & R(-2) & R(-4) & R(-6) & R(-9)\\ R(2) & R(0) & R(-2) & R(-4) & R(-7)\\ R(4) & R(2) & R(0) & R(-2) & R(-5)\\ R(6) & R(4) & R(2) & R(0) & R(-3)\\ R(9) & R(7) & R(5) & R(3) & R(0) \end{array}\right]+\sigma_{n}^{2}I$$

We reconstruct R into $R_{T}$ with the dimension 10×10:

(16) $$R_{T}=\;\left[\begin{array}{ccccccc} R(0) & 0 & R(-2) & \cdots & R(-7) & 0 & R(-9)\\ 0 & R(0) & 0 & \cdots & R(-6) & R(-7) & 0\\ R(2) & 0 & R(0) & \cdots & R(-5) & R(-6) & R(-7)\\ \vdots & \vdots & \vdots & \ddots & \vdots & \vdots & \vdots \\ R(7) & R(6) & R(5) & \cdots & R(0) & 0 & R(-2)\\ 0 & R(7) & R(6) & \cdots & 0 & R(0) & 0\\ R(9) & 0 & R(7) & \cdots & R(2) & 0 & R(0) \end{array}\right]+\sigma_{n}^{2}I$$

The estimation of the R is obtained with *L* snapshots:

(17) $$\hat{R}=\frac{1}{L}\sum_{l=1}^{L}X(t_{l})X^{H}(t_{l})$$

${\hat{R}}_{T}$ can be obtained by reconstructing the $\hat{R}$. Based on the matrix completion theory, zero elements in $R_{T}$ can be filled to obtain the target matrix $R_{c}$. According to [29], the matrix $R_{c}$ can be obtained by the following optimization problem:

(18) $$\begin{array}{ll} \mathrm{minimize}\; & \mathrm{rank}(R_{c})\\ \mathrm{subject}\;\mathrm{to}\; & P\;\cdot \;{\hat{R}}_{T}=P\;\cdot \;R_{c} \end{array}$$

where $R_{c}$ is the target matrix, and $\mathrm{rank}(R_{c})\;$ is the rank of $R_{c}$. P denotes the projection matrix, and the element of P is:

(19) $$P(i,j)=\{\begin{array}{cc} 1, & \mathrm{if}\;i-j=p_{u},\\ 0, & \mathrm{otherwise}, \end{array}$$

The rank function is a non-convex function, so the model (18) is non-deterministic polynomial (NP-hard) problem. The nuclear norm is an approximation of the rank norm, and model (18) can be appropriately convex relaxed:

(20) $$\begin{array}{ll} \mathrm{minimize} & {\left\|R_{c}\right\|}_{*}\\ \mathrm{subject}\;\mathrm{to} & P\;\cdot \;{\hat{R}}_{T}=P\;\cdot \;R_{c}\; \end{array}$$

where ·  represent Hadamard product. To address the optimization problem of model (20), we employ SVT algorithm to perform matrix completion. The model (20) is thus converted to an approximation problem:

(21) $$\begin{array}{ll} \mathrm{minimize} & \mu {\left\|R_{c}\right\|}_{*}+\frac{1}{2}{\left\|R_{c}\right\|}_{F}^{2}\\ \mathrm{subject}\;\mathrm{to} & P\;\cdot \;{\hat{R}}_{T}=P\;\cdot \;R_{c}\; \end{array}$$

The optimal solution of (21) approximates the solution of (20) as μ→∞. Therefore, selecting a larger μ can achieve convex relaxation. For the optimization problem shown in (21), the solution of the SVT method is as follows:

(22) $$\{\begin{array}{c} R_{c}^{k}=D_{\mu }(Y^{k-1}),\\ Y^{k}=Y^{k-1}+\delta_{k}P\;\cdot \;({\hat{R}}_{T}-R_{c}^{k}) \end{array}$$

where Y is intermediate matrix. The key property here is that for large values of μ, the sequence {$R_{c}^{k}$} of model (22) converges to a solution which very nearly minimizes (21). $\delta_{k}$ is the iteration step. From [26], if $0<\delta_{k}<2$, the matrix completion problem of (22) can be guaranteed to converge. There is a large literature on ways of selecting a step size, but for simplicity, we shall use step sizes that are independent of the iteration count; that is $\delta_{k}=\delta$, for k=1, 2, …. As mentioned above, convergence for the completion problem is guaranteed provided that 0<δ<2. However, this selection is too conservative and the convergence rate is very slow. In our experiments, we select a constant $\delta =1.2\frac{n^{2}}{L}$ that not related to the number of iterations, where n is the dimension of the square matrix $R_{c}$, and L is the number of snapshots. $D_{\mu }$ is the soft-thresholding operator. It is defined as follows:

(23) $$D_{\mu }(Y)≜UD_{\mu }(\Sigma )V^{*},\;D_{\mu }(\Sigma )=diag\left(\left\{{\left(\sigma_{i}-\mu \right)}_{+}\right\}\right)$$

where ${\left(\sigma_{i}-\mu \right)}_{+}=\max(0,\sigma_{i}-\mu )$, $\sigma_{i}$ is the singular value of Y. In the (22) kth iteration, $z_{k}$ is the number of singular values of $Y^{k-1}$. The calculation process is as follows:

(24) $$r_{k}=\mathrm{rank}(Y^{k-1})\;,\;z_{k}=r_{k-1}+1$$

If the calculated singular value is less than μ, then the $z_{k}$ is the right selection. Otherwise, repeatedly increase the $z_{k}$ by a predefined integer h until singular values fall below μ. The iteration is natural to stop, when the following conditions are met:

(25) $$\frac{{\left\|P\;\cdot \;(R_{c}^{k}-{\hat{R}}_{T})\right\|}_{F}}{{\left\|P\;\cdot \;({\hat{R}}_{T})\right\|}_{F}}\leq \varepsilon$$

where ε is a fixed tolerance, e.g., 10^−4^. The detailed steps of the proposed SVT-based DOA estimation algorithm are summarized in Table 1.

## 4. Simulation Results

In this section, simulation results are performed to illustrate the superiority of our methods. The angle search interval of CO-MUSIC is set to 0.1° and the grid interval of CO- Lasso [24] is set to 0.1°.

### 4.1. Spatial Spectrum

In the first example, simulation results are presented when there are more sources than sensors. The pair of coprime integers are chosen as M=5 and N=4. The intact coprime array consists of 2M+N−1=13 sensors. We assume that there are 18 far-field narrowband sources with incident angles of −57.3°, −50.8°, −44.4°, −39.3°, −32.1°, −26.8°, −18.7°, −11.2°, −4.5°, 3.1°, 9.4°, 14.8°, 21.3°, 28.4°, 36.2°, 41.9°, 48.3°, 55.4°. The snapshot number is 300 with SNR = 0 dB.

**Figure 4:**
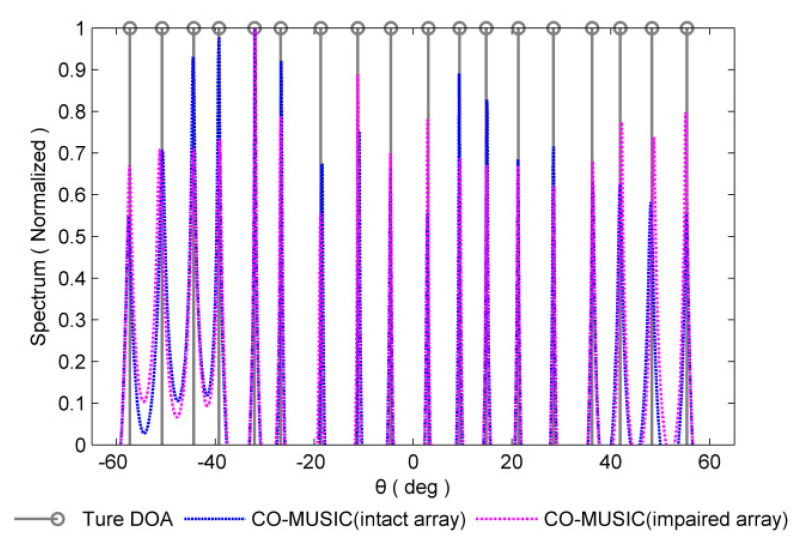
Normalized spectra of the coarray MUSIC (CO-MUSIC) in redundant sensor failures scenario and intact array scenario are considered. The number of snapshots is 300 and SNR = 0 dB.

For redundant sensor failures, take the {4d,12d,20d} position sensors failure in Figure 3 as an example, and the set of consecutive lag positions is {0,±1d,⋯,24d}, which is the same as the consecutive lags in the intact coprime array. The CO-MUSIC algorithm is employed to perform simulations on intact array and impaired array respectively in Figure 4. It can be seen that the consecutive lags in the redundant sensor failure scenario have not changed. By employing MUSIC for DOA estimation, the original estimated performance can be basically restored.

**Figure 5:**
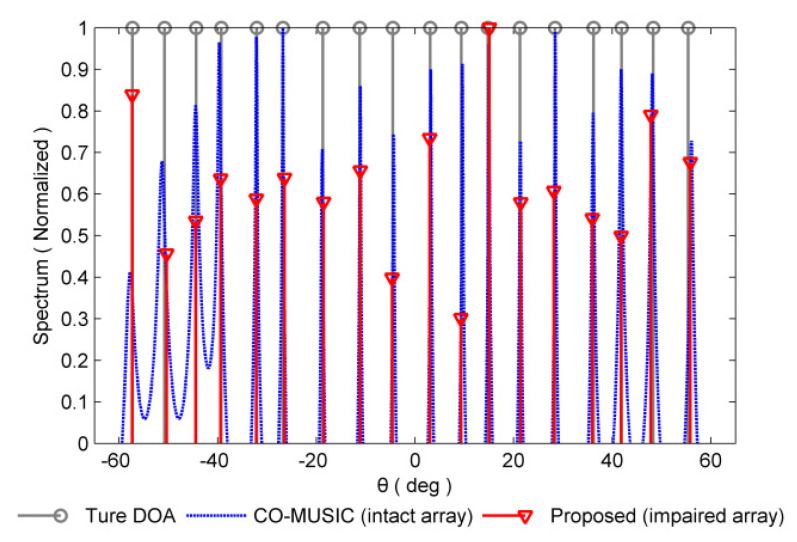
Normalized spectra of the proposed method in non-redundant sensor failures scenario and the CO-MUSIC in intact array scenario are considered. The number of snapshots is 300 and SNR = 0 dB.

For non-redundant sensor failures, take the {5d,20d,24d} position sensors failure in Figure 3 as an example, and the set of consecutive lag positions is {0,±1d,⋯,±8d}. In this failure scenario, the CO-MUSIC algorithm is completely invalid and cannot perform DOA estimation on 18 sources. Therefore, we use the proposed algorithm for DOA estimation as shown in Figure 5. Compared with the CO-MUSIC performance of intact array, it can be seen that under the non-redundant sensor failure scenario, the proposed algorithm can give good estimation result.

### 4.2. RMSE Versus SNR and Number of Snapshots

In the second example, we simulate the DOA estimation accuracy of CO-MUSIC, CO-Lasso and proposed method. We use the root mean square error (RMSE) as a performance indicator. RMSE is defined as:

(26) $$\mathrm{RMSE}=\sqrt{\frac{1}{\mathrm{JQ}}\sum_{j=1}^{J}\sum_{q=1}^{Q}{\left({\hat{\theta }}_{q}(j)-\theta_{q}\right)}^{2}}$$

where J is the number of Monte Carlo trials, Q is the number of sources, $\theta_{q}$ is the true DOA of the qth source and ${\hat{\theta }}_{q}(j)$ is the estimate of $\theta_{q}$ for the jth trial.

**Figure 6:**
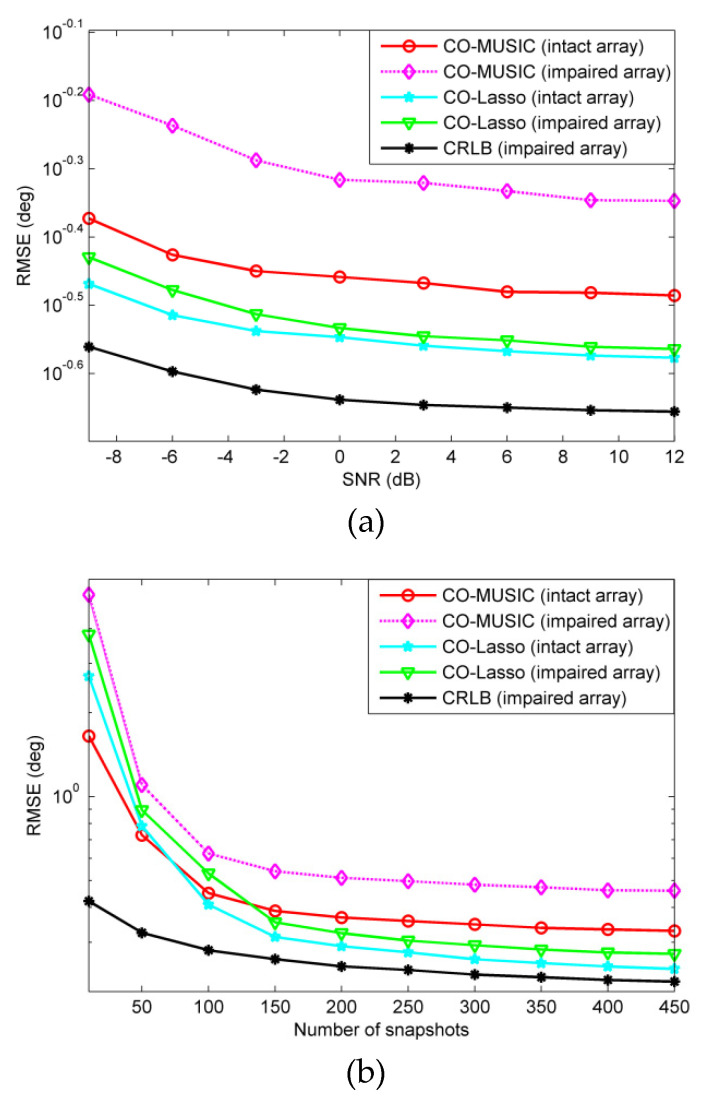
The scenario of redundant sensor failures, the root mean square error (RMSE) performance comparison of CO-MUSIC and CO-Lasso with 500 Monte Carlo trials and 18 sources considered. (**a**) RMSE versus SNR with *L* = 300 and (**b**) RMSE versus the number of snapshots with SNR = 0 dB.

**Figure 7:**
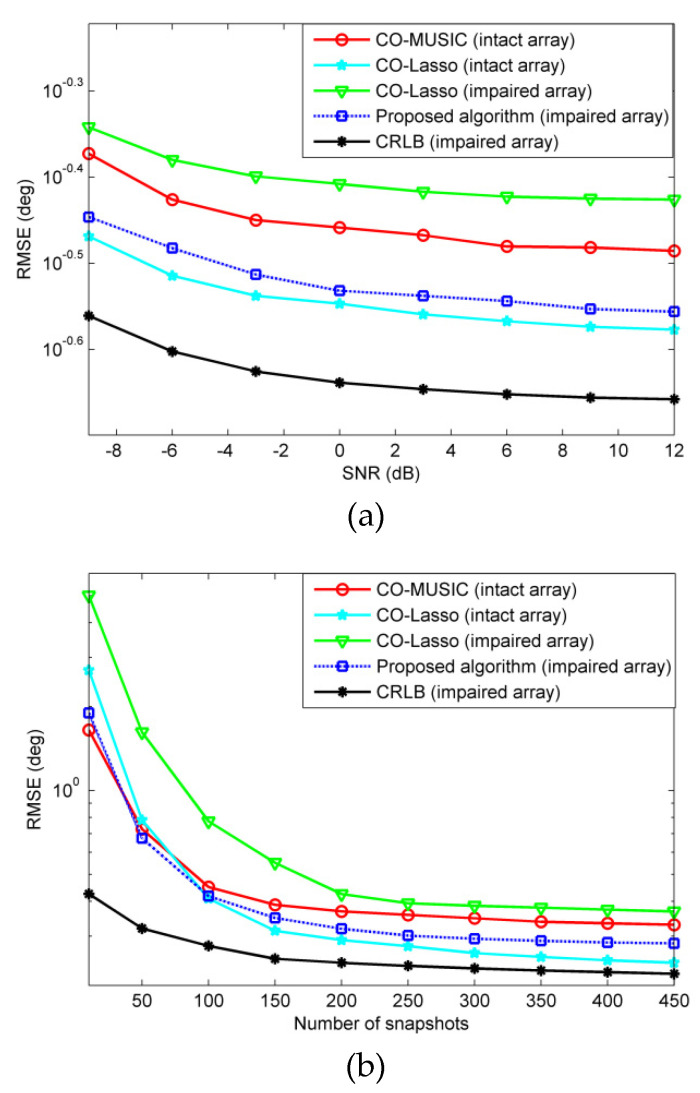
The scenario of non-redundant sensor failures, the RMSE performance comparison of CO-MUSIC, CO-Lasso, and the proposed method with 500 Monte Carlo trials and 18 sources considered. (**a**) RMSE versus SNR with *L* = 300 and (**b**) RMSE versus the number of snapshots with SNR = 0 dB.

We assume that there are 18 far-field narrowband sources with incident angles of −57.3°, −50.8°, −44.4°, −39.3°, −32.1°, −26.8°, −18.7°, −11.2°, −4.5°, 3.1°, 9.4°, 14.8°, 21.3°, 28.4°, 36.2°, 41.9°, 48.3°, 55.4°. We simulated the RMSEs under two failure scenarios, performing 500 independent Monte Carlo trials. In order to compare RMSE against a benchmark, the Cramér-Rao Lower Bound (CRLB) for the impaired coprime array is plotted for reference. When a few sensors of the co-prime array fail, the remaining non-failed sensors are equivalent to form a new sparse array. We use the CRLB in [30] as a benchmark in Figure 6 and Figure 7.

For redundant sensor failures, take the {4d,12d,20d} position sensors failure in Figure 3 as an example. Figure 6 is RMSE versus SNR and snapshots. It is clear that the estimation accuracy of CO-MUSIC (impaired array) is significantly lower than that of CO-MUSIC (intact array) under the scenario of redundant sensor failures. The estimated performance of CO-Lasso (impaired array) is better than that of CO-MUSIC (intact array). Therefore, for redundant sensor failure scenarios, the CO-Lasso can recover estimated performance. Note that in the scenario of redundant sensor failures, the performance of CO-MUSIC is worse than that of CO-Lasso, but the computational complexity is less, thus CO-MUSIC still has application value.

For non-redundant sensor failures, take the {5d,20d,24d} position sensors failure in Figure 3 as an example. Figure 7 is RMSE versus SNR and snapshots. In this failure scenario, the consecutive lags position of the array is {0,±1d,⋯,±8d}, and CO-MUSIC is completely invalid and cannot perform DOA estimation on 18 sources. Figure 7 shows that the estimation error of CO-Lasso (impaired array) is larger than that of CO-MUSIC (intact array), and the estimated performance cannot be recovered. The examples of non-redundant sensor failures and redundant sensor failures both fail 3 sensors, but the information received by non-redundant sensors is more important than redundant sensors. Therefore, CO-Lasso can recover DOA estimation performance in the scenario of redundant sensor failures, but poor performance in non-redundant sensor scenarios. The proposed algorithm fills the holes and uses all the lags in the difference coarray. Compared with CO-Lasso, the proposed algorithm has obvious advantage in DOA estimation performance, and can recover the performance in non-redundant sensor failure scenarios.

The virtual array-based CRLB is presented in Figure 6 and Figure 7 as the reference. It is observed from Figure 6 and Figure 7 that when there are more sources than the number of sensors, with the increase of the SNR, the CRLB gradually converges to a constant rather than keeps decreasing linearly. This is the typical *saturation* behavior [31].

### 4.3. Angular Resolution

In the third example, we simulate the angular resolution of each algorithm. If both $\left|{\hat{\theta }}_{1}-\theta_{1}\right|$ and $\left|{\hat{\theta }}_{2}-\theta_{2}\right|$ are smaller than $\left|\theta_{1}-\theta_{2}\right|/2$, the two sources are considered to be successfully resolved [32], where $\theta_{1}$ and $\theta_{2}$ are true DOAs, ${\hat{\theta }}_{1}$ and ${\hat{\theta }}_{2}$ are the DOA estimates. We set $\theta_{1}=30{}^{\circ}$ and $\theta_{2}=\theta_{1}+∆\theta$, where ∆θ varies from 0.1° to 2° with a step of 0.1°. We set $\theta_{1}=30{}^{\circ}$ and $\theta_{2}=\theta_{1}+∆\theta$, where ∆θ is a small variable that varies from 0.1 ° to 2 ° in steps of 0.1 °. Note that the probability of resolution is different for different directions and decreases with the angular separation of the sources to be resolved from the array’s broadside. We randomly choose a direction range and compare the probability of resolution of several methods in this direction range before and after sensor failures. In our simulation, we choose $\theta_{1}=30{}^{\circ}$ and $\theta_{2}=\theta_{1}+∆\theta$. The purpose is to show that the proposed method can restore the angle resolution performance better than other methods under the condition of sensor failures.

**Figure 8:**
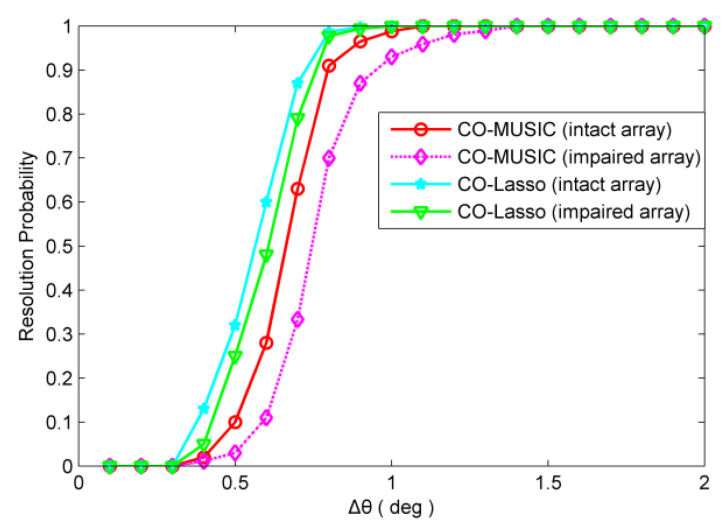
The scenario of redundant sensor failures, resolution probability versus ∆θ. Two sources are set to $\theta_{1}=30{}^{\circ}$ and $\theta_{2}=\theta_{1}+∆\theta$. The snapshot number is 300 with SNR = 0 dB.

**Figure 9:**
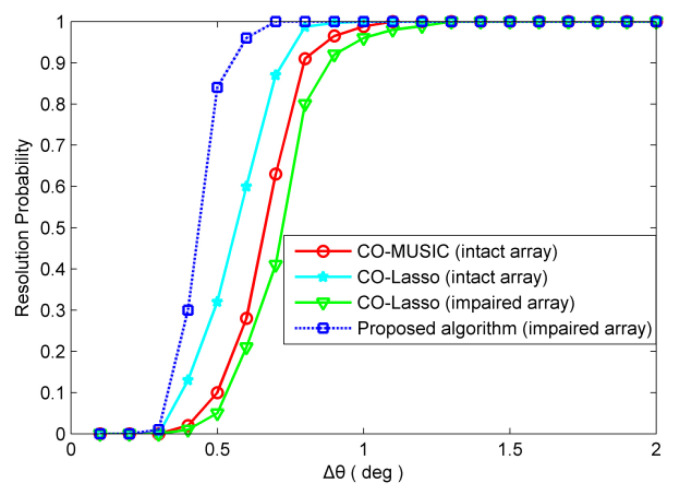
The scenario of non-redundant sensor failures, resolution probability versus ∆θ. Two sources are set to $\theta_{1}=30{}^{\circ}$ and $\theta_{2}=\theta_{1}+∆\theta$. The snapshot number is 300 with SNR = 0 dB.

For redundant sensor failures, take the {4d,12d,20d} position sensors failure as an example. For non-redundant sensor failures, take the {5d,20d,24d} position sensors failure as an example. The number of snapshots is 300 and SNR = 0 dB. The resolution probability obtained from 500 independent Monte Carlo trials are depicted in Figure 8 and Figure 9. As shown in Figure 8, in the scenario of redundant sensor failures, CO-Lasso (impaired array) has higher angular resolution than CO-MUSIC (intact array), and CO-MUSIC (impaired array) has the lowest angular resolution. It is clear from Figure 9 that in the scenario of redundant sensor failures, the angular resolution of CO-Lasso is degraded. The proposed algorithm has significant performance advantages in angular resolution.

## 5. Conclusions

The coarray structure of the coprime array is sensitive to sensor failures. Once the sensor fails, the impact of failure sensors on the difference coarray may decrease the DOFs and cause direction finding failure. In view of this problem, firstly, we advance a detection strategy to diagnose the coprime array. Furthermore, the sensor failure scenarios are divided into two scenarios. For redundant sensor failures, the structure of difference coarray remains unchanged, and the CO-MUSIC algorithm is applicable. For non-redundant sensor failures, we reconstruct the covariance matrix into a matrix with zero elements, and employ the SVT algorithm to perform matrix completion, thereby filling the holes. 

## Figures and Tables

**Table 1:** The proposed singular value thresholding- (SVT) based direction of arrival (DOA) estimation algorithm.

Input: Sampled set Ω, received data vector x(t), mapping matrix P, step size δ, fixed tolerance ε, parameter μ, increment h, and maximum iteration count $k_{\max}.$ Output: DOA estimation results.
Compute the sample covariance matrix $\hat{R}$; Reconstruct and extend $\hat{R}$ into a high-dimensional ${\hat{R}}_{T}$ with the dimension (2MN−N+1)×(2MN−N+1); Set $Y^{0}=k_{0}\delta P\;\cdot \;({\hat{R}}_{T})$, $\frac{\mu }{\delta {\left\|P\;\cdot \;({\hat{R}}_{T})\right\|}_{2}}\in (k_{0}-1,k_{0}]$,$r_{0}=0$; **for** k=1 **to** $k_{\max}$; Set $z_{k}=r_{k-1}+1$; **repeat** Compute ${\left[U^{k-1},\Sigma^{k-1},V^{k-1}\right]}_{z_{k}}$; Set $z_{k}=z_{k}+h$; **until** $\delta_{z_{k}-h}^{k-1}\leq \mu$; Set $r_{k}=\max\left\{j:\sigma_{j}^{k-1}>\mu \right\}$; Set $R_{c}^{k}=\sum_{j=1}^{r_{k}}\left(\sigma_{j}^{k-1}-\mu \right)u_{j}^{k-1}v_{j}^{k-1}$; **if** ${\left\|P\;\cdot \;(R_{c}^{k}-{\hat{R}}_{T})\right\|}_{F}/{\left\|P\;\cdot \;({\hat{R}}_{T})\right\|}_{F}\leq \varepsilon$ **then** break; Set $Y_{ij}^{k}=\left\{ \begin{array}{ll} 0 & \mathrm{if}\;(i,j)\notin \Omega \\ Y_{ij}^{k-1}+\delta \left({\left({\hat{R}}_{T}\right)}_{ij}-{\left(R_{c}^{k}\right)}_{ij}\right) & \mathrm{if}\;(i,j)\in \Omega \end{array}\right.$; **end** for k; Set $R_{c}^{opt}=R_{c}^{k}$; Employ root-MUSIC for DOA estimation.
